# Terahertz all-silicon metasurfaces with off-axis bifocal characteristics for polarization detection

**DOI:** 10.1515/nanoph-2023-0277

**Published:** 2023-06-22

**Authors:** Hui Li, Shouxin Duan, Chenglong Zheng, Hang Xu, Jie Li, Chunyu Song, Fan Yang, Wei Shi, Yating Zhang, Yun Shen, Jianquan Yao

**Affiliations:** Ministry of Education, School of Precision Instruments and Opto-Electronics Engineering, Key Laboratory of Opto-Electronics Information Technology (Tianjin University), Tianjin University, No. 92 WeiJin Road, Tianjin 300072, China; Department of Physics, School of Physics and Materials Science, Nanchang University, Nanchang 330031, China; Information Materials and Device Applications Key Laboratory of Sichuan Province, Chengdu University of Information Technology, 610225, Chengdu, China

**Keywords:** all-silicon metasurface, full-Stokes parameter, polarization detection, polarization multiplexing

## Abstract

Functional devices for terahertz (THz) polarization detection in transmission mode are highly desired in integrated applications, but traditional polarization measurement systems are bulky and highly cost. The combination between all-silicon metasurfaces and focused beams carrying polarization information has offered a new opportunity for miniaturized polarization detection behavior. Here, we investigate and experimentally demonstrate a new scheme for realizing efficiently miniaturized polarization detection behavior based on the polarization multiplexing encoding technique. The full-Stokes parameter matrix of the incident polarization state can be reconstructed in a single snapshot by using a microprobe to record, pixel by pixel, the complex amplitude information contained in a pre-designed plane. Subsequently, the polarization detection capability of the proposed design principle is evaluated using random polarization states defined on the surface of a standard Poincaré sphere (PS). Such a scheme offers potential applications for the development of compact photonic meta-platforms for polarization detection in transmission mode, being highly favored in polarization high-resolution imaging, remote sensing, and THz communications.

## 1 Introduction

As the key parameters that determine the fundamental properties of electromagnetic (EM) waves, frequency, amplitude, phase, and polarization are also the representative basis for proving that free-space EM waves are transverse waves [1–3]. The polarization state, characterized by the direction of oscillation of the electric field, can effectively enhance the interaction between light and matter. A variety of applications have been reported for the manipulation of polarization states, for example, medicine [4], microscopy [5], and remote sensing [6]. Therefore, not only is it possible to generate the desired polarization state using a polarizer, but the detection of the polarization state also plays an important role in photonic applications [7–9]. The full-Stokes parameter matrix is commonly employed to evaluate the incident polarization state and can be determined using the intensity difference among the different polarization components [10–12]. However, the peculiarities of the THz band, such as the enormous insertion loss, make it impossible to measure the S-parameters belonging to the full Stokes matrix using the conventional approach [13]. Therefore, there is an urgent requirement to develop a polarization detection scheme that is suitable and efficient for the THz band. On the other hand, due to the relatively long wavelength of THz waves, the conventional polarization detection-related functional devices have a large size, which weakens the process of system integration and miniaturization [14].

Metasurface, as a novel two-dimensional planar structure, has a high degree of design freedom for each meta-atom in the array [15–18]. The all-dielectric meta-platform working in transmission mode, for example, usually has the advantages of high damage threshold, ultra-thin size, high modulation efficiency and large response bandwidth, which makes it uniquely advantageous for the integration of THz polarization-related functional devices [19–21]. To the best of our knowledge, a completed polarization state can be determined in terms of major axis, ellipticity, and handedness [22, 23]. In fact, the three parameters mentioned above are also the key components used to draw polarization ellipses with visualization properties. The presence of the parameter *ψ* describes the angle of the major axis of the polarization ellipse with respect to the horizontal direction, specifying its orientation. While the ellipticity *η* is described by the intensity ratio of the left-handed circular polarization (LCP) and right-handed circular polarization (RCP) components. In other words, *η* = 1 and *η* = 0 represent the LCP (RCP) and LP, respectively. For the handedness, it can be readily obtained from the recorded complex amplitude information.

In the THz band, to determine the polarization state of the pulse signal emitted by a THz time-domain spectrometer (TDS), a coherent method is usually used for polarization detection [24]. Wang et al. [25]. reported a reflective metasurface with four focal points for THz polarization detection in theory, however, there are several difficulties in the experimental verification. Nowack et al. [26], experimentally demonstrated an all-silicon metasurface operating at 118.8 μm. Applying a typical polarization-separated meta-platform with a hexagonal lattice, focal points for characterizing the incident polarization state were generated at different spatial coordinates. However, the designed complex optical path assembly is not applicable to the polarization detection behavior at longer wavelengths. Recently, Jiang et al. [27]. applied a Bessel vortex beam generated based on polarization multiplexing technique to achieve the detection of polarization states of continuous THz waves. Although the interferometric spot obtained along the propagation direction does not determine the key parameters composing the polarization ellipse one by one, the application of the polarization multiplexing technique provides a reference for realizing the polarization detection of pulsed THz beams. Despite significant efforts have been devoted to designing wavefront-assisted THz metasurfaces, to our knowledge, no experimental result has been demonstrated in the THz range for arbitrary incident polarization states using multiplexing coding techniques. In Table 1, we summarize the reported polarization detection performance in the THz range.

**Table 1:** Scheme for polarization detection in the THz range.

Target polarization type	Working mode	Encoding method	Number of foci	Ref.
Arbitrary polarization states	Reflection	PB phase	4	[25]
Circular polarization states	Transmission	Dynamic and PB phases	2	[36]
Arbitrary polarization states	Transmission	Dynamic phase	6	[26]
Linear polarization states	Transmission	Dynamic phase	–	[27]
Linear polarization states	Transmission	Dynamic phase	1	[20]
Arbitrary polarization states	Transmission	Dynamic and PB phases	2	This work

**Figure 1:**
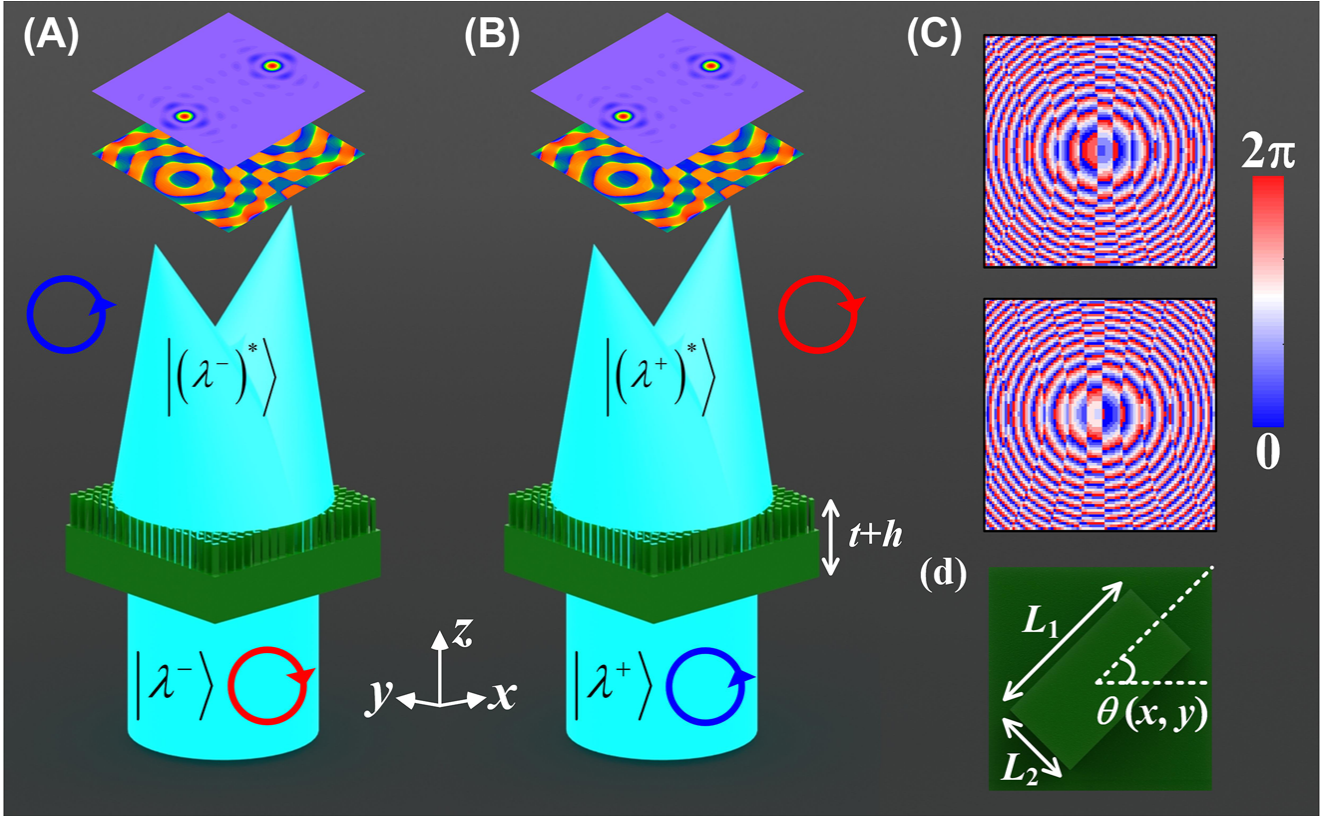
Schematic diagram of the THz polarization detection approach based on the polarization multiplexing coding technique. (A) Electric field and phase distribution with off-axis bifocal characteristics for RCP incidence. (B) Electric field and phase distribution with off-axis bifocal characteristics for LCP incidence. (C) Phase profiles embedded within the orthogonal CP channels using polarization multiplexing techniques. (D) Top view of the basic building block of the rectangular configuration is presented, and the key structural parameters include *L*_1_, *L*_2_ and the in-plane rotation angle *θ* (*x*, *y*), respectively.

In this work, an all-silicon metasurface based on polarization multiplexing encoding technique is demonstrated (in Figure 1A and B), combining the matrix of full-Stokes component, and using polarization ellipse as a key parameter, vividly realizing one-to-one mapping of incident polarization states with far-field images. The encoding technique with decoupling effect imparts phase profiles with off-axis bifocal characteristics to the LCP and RCP channels, respectively, as shown in Figure 1C. By extracting the complex amplitude information at different positions on the pixelated focal plane, the key parameters belonging to the full Stokes matrix as well as the polarization ellipse can be reconstructed continuously. In fact, the phase difference embedded within the orthogonal CP channels guarantees that the polarization states reconstructed at off-axis bifocal points can be verified against each other. Besides, the application of the phase encoding technique with extended focal length in the orthogonal circular polarization (CP) channel guarantees a good agreement between experimental and simulation results. Thus, the proposed scheme opens a new window for future THz polarization detection behavior and breathes new life into the development of novel, ultra-compact and high-performance meta-optoelectronic devices.

## 2 Design and methods

**Figure 2:**
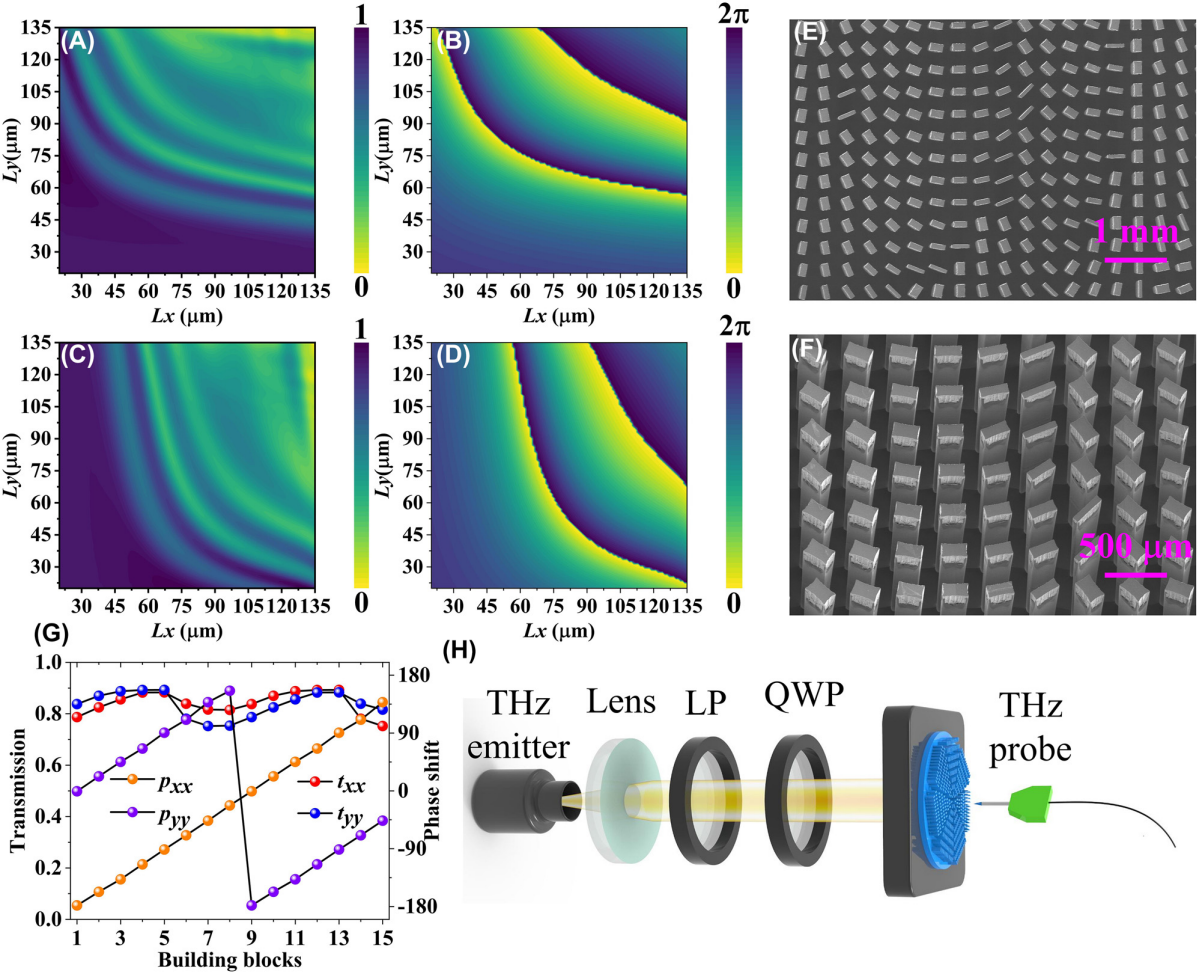
The fundamental features of meta-atoms with anisotropy. The database established by a wide range of parametric sweeps, which includes (A) transmission amplitudes at *x*-polarized incidence, (B) transmission phase-maps at *x*-polarized incidence, (C) transmission amplitudes at *y*-polarized incidence, and (D) transmission phase-maps at *y*-polarized incidence. Local scanning electron microscope (SEM) photo of the fabricated sample with scale bars of (E) 1 mm, and (F) 500 μm. (G) The amplitude and phase response of the selected 15 meta-atoms that satisfy the spin decoupling condition. (H) The optical path of the near-field THz scanning system equipped with a probe.

A schematic diagram of the designed all-silicon metasurface based on CP multiplexing encoding technique is shown in Figure 1, which can produce the off-axis tightly focused beams carrying specific polarization information in the focal plane [28–30]. For example, THz plane waves with RCP states can induce the proposed meta-platform to produce tiny focal spots at spatial coordinates of (−1000, 0, 5000) and (1000, 0, 5000), as shown in Figure 1A. It should be emphasized that arbitrary polarization state can be expressed as a superposition of LCP and RCP components. Thus, the vertically incident LCP beams will be converged to the same spatial coordinates, as shown in Figure 1B. In order to achieve the desired function shown in Figure 1, the phase distribution Φ_*RL*_ and Φ_*LR*_ within the orthogonal circularly polarized (CP) channel should satisfy that [31–33],

(1) $$\left\{\begin{array}{c} \Phi_{LR}^{1}\left(r,\varphi \right)=\frac{k\cdot r_{1}^{2}}{2\left(f+\Delta f\cdot r_{1}^{2}/R^{2}\right)}+\varphi_{LR}^{1},\Phi_{LR}^{2}\left(r,\varphi \right)=\frac{k\cdot r_{2}^{2}}{2\left(f+\Delta f\cdot r_{2}^{2}/R^{2}\right)}+\varphi_{LR}^{2}\\ \Phi_{RL}^{1}\left(r,\varphi \right)=\frac{k\cdot r_{1}^{2}}{2\left(f+\Delta f\cdot r_{1}^{2}/R^{2}\right)}+\varphi_{RL}^{1},\Phi_{RL}^{2}\left(r,\varphi \right)=\frac{k\cdot r_{2}^{2}}{2\left(f+\Delta f\cdot r_{2}^{2}/R^{2}\right)}+\varphi_{RL}^{2} \end{array}\right.$$

(2) $$\Phi_{tot}=\arg\left\{\overset{2}{\underset{n=1}{\sum }}\left[\exp\left(i\Phi_{LR}^{n}\right)+\exp\left(i\Phi_{RL}^{n}\right)\right]\right\}$$

where *k* = 2*π*/*λ* represents the wave vector in free space, *λ* denotes the wavelength corresponding to 0.8 THz, $\varphi_{LR}^{1}=\varphi_{RL}^{1}=0$ and $\varphi_{LR}^{2}=-\varphi_{RL}^{2}=\pi /2$ indicate the initial phase difference within two CP channels, $r_{1}=\sqrt{{\left(x+1000\right)}^{2}+y^{2}}$ , $r_{2}=\sqrt{{\left(x-1000\right)}^{2}+y^{2}}$ denote the radius of the two polar coordinates presenting an off-axis distribution, respectively, and Φ_tot_ denotes the total phase distributions calculated to generate the metasurface [34]. It is worth noting that the adoption of the phase profile with extended depth of focus effectively improves the focusing quality of the resulting beam, thus reducing the error in the detection of the incident polarization. The focal lengths were set to *f* = 5 mm and the extended focal range was set to Δ*f* = 0.5 mm. Benefitting from the fundamental design principle of circular birefringence, the metasurface introduces spin-dependent differences in the axial component of the incident THz wave. Not only that, the proposed spin-polarization multiplexing coding scheme can be applied to arbitrary wavebands for reconstructing the full Stokes parameter matrix of the incident polarization state. Detailed simulation results of the generalized design can be found in Supplementary Section 1. The phase profile of the converging beam with tight focusing characteristics generated according to Equation (1) is shown in Figure 1C. By manipulating the initial phase difference within the orthogonal CP channels, the proposed design can reconstruct the vibrational trajectory of an arbitrary incident polarization state. The all-dielectric meta-atoms with high aspect ratio (AR) are introduced as the key elements composing the metasurface, as shown in Figure 1D. The key parameters that formed the silicon column were denoted as *L*_1_, *L*_2_, and *θ* (*x*, *y*), respectively. Here, *L*_1_ and *L*_2_ determine the dynamic phase distributions while *θ* (*x*, *y*) induces the geometric phase responses. Figure 2A–D illustrate the parameter library of the meta-atoms constructed by changing *L*_1_ and *L*_2_ in the simulation. CST MICROWAVE STUDIO is applied to simulate truncated waveguides with periodic boundary conditions, details of which can be found in Supplementary Section 2. The meta-atoms with anisotropy provide potential opportunities for spin-dependent polarization manipulation. In other words, the selected meta-atoms satisfying the spin decoupling condition operate with properties similar to half-wave plates (HWPs) with significant polarization flipping behavior. It is worth mentioning that the structural parameters of the meta-atoms as a function of the transmitted amplitude and phase shift are generated under orthogonal linearly polarized (LP) illumination. Moreover, the all-dielectric meta-atoms presenting a rectangular configuration have oscillatory and efficient transmission behavior due to the Fabry–Perot resonance effect, as mentioned in Supplementary Section 3. According to the limitations of spin decoupling, the phase relations between the CP components and the expected LP components can be expressed as [34, 35],

(3) $$\left\{\begin{array}{c} \phi_{xx}=\left(\phi_{\mathrm{LCP}}+\phi_{\mathrm{RCP}}\right)/2\\ \phi_{yy}=\left(\phi_{\mathrm{LCP}}+\phi_{\mathrm{RCP}}\right)/2-\pi \\ \theta =-\left(\phi_{\mathrm{LCP}}-\phi_{\mathrm{RCP}}\right)/4 \end{array}\right.$$

where *ϕ*_*xx*_ and *ϕ*_*yy*_ represent the phase distributions under *x*- and *y*-polarized incidence, *ϕ*_*LCP*_, and *ϕ*_*RCP*_ denote the phase profiles under LCP and RCP illumination. The selected structural parameters of the basic meta-atoms operating at 0.8 THz can be found in the Supplementary Section 4, achieving full 2π phase control. Samples based on commercial high-resistance silicon wafers were fabricated by employing ICP (Inductively Coupled Plasma) etching technique [33–37]. In addition, the desired sample was fabricated using a 4-inch silicon wafer with a thickness of 1 mm, and the height *h* of the dielectric column was about 400 μm. As shown in Figure 2E and F, scanning electron microscopy (SEM) images of the samples taken at different scales all show steep sidewalls with tolerable fabrication errors. In addition, the specific steps of the sample fabrication session are described in detail in Supplementary Section 5. It is worth mentioning that the rectangular silicon pillars composing the fabricated samples exhibit high transmission efficiency under the illumination of different LP plane waves supported by Fabry–Perot resonance. Moreover, the operating states of the selected 15 basic building blocks strictly obey the HWP, as shown in Figure 2G.

The working principle of the metasurfaces with off-axis bifocal characteristics for THz polarization detection is illustrated in Figure 1A and B. The pixelated focal plane imparts an extraordinary means of extracting the individual polarization components at arbitrary polarization incidence. Subsequently, the standard algorithm can be applied to reconstruct the full Stokes parameter matrix of the incident randomly polarization states. Extracting the *E*_*x*_- and *E*_*y*_-components from the total electric field distribution allows the Stokes parameters to be readily calculated as [11, 13],

(4) $$S=\left[\begin{array}{c} S_{0}\\ S_{1}\\ S_{2}\\ S_{3} \end{array}\right]=\left[\begin{array}{c} E_{x}^{2}+E_{y}^{2}\\ E_{x}^{2}-E_{y}^{2}\\ 2E_{x}E_{y}\cos\left(\delta_{y}-\delta_{x}\right)\\ 2E_{x}E_{y}\sin\left(\delta_{y}-\delta_{x}\right) \end{array}\right]$$

where *δ*_*x*_ and *δ*_*y*_ denotes the phase difference between the two polarization components, *E*_*x*_ and *E*_*y*_ represent the amplitude of the orthogonal LP channels, respectively. Therefore, the polarization state of the incident THz beam can be determined by extracting the complex amplitude of the pixel at the pre-defined coordinates. It is worth mentioning that the pixelated complex amplitudes extracted from the focal plane can be utilized to plot polarization ellipses, enabling further visualization of the incident polarization. The standard polarization ellipse can be defined as [38, 39],

(5) $$\frac{E_{x}^{2}}{E_{0x}^{2}}+\frac{{E_{y}}^{2}}{{E_{0y}}^{2}}-2\frac{E_{x}}{E_{0x}}\frac{E_{y}}{E_{0y}}\cos\left(\delta_{y}-\delta_{x}\right)=\sin^{2}\left(\delta_{y}-\delta_{x}\right)$$

## 3 Results and discussions

**Figure 3:**
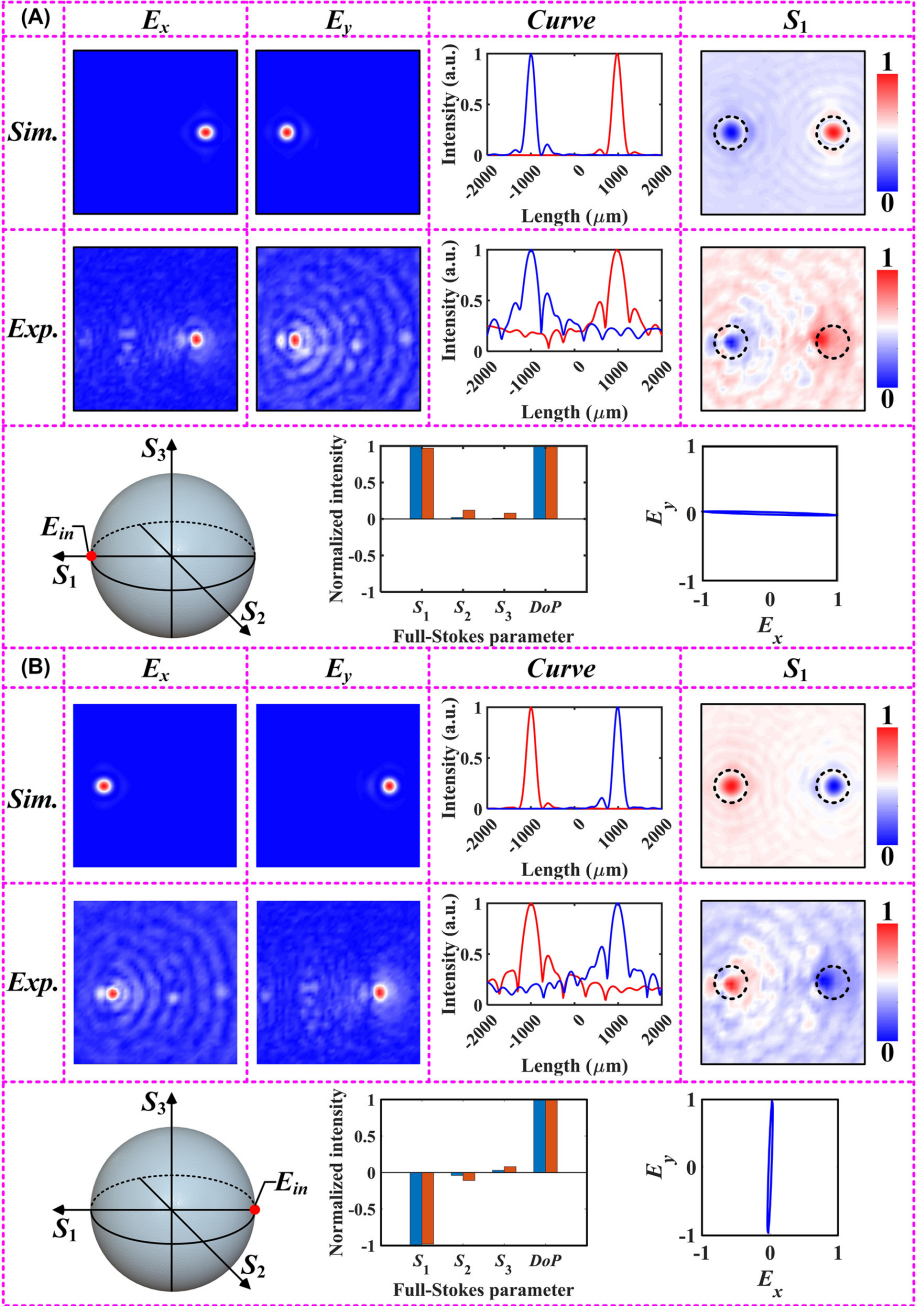
Simulated and experimental results of the fabricated samples at different polarization incidences. This includes the electric field distribution obtained from direct measurements, the normalized cross-sectional intensity profile, and the parameter *S*_1_ obtained by data processing. In addition, the incident polarization is represented using a standard PS, and the full-Stokes parameter matrix of the incident polarization and the visualized polarization ellipse are reconstructed using the complex amplitude information recorded in the focal plane. (A) Under *x*-polarized incidence, and (B) under *y*-polarized incidence.

Utilizing a THz near-field scanning system as schematically shown in Figure 2H. Apparently, the system generates a pulsed THz beam for vertically illuminating the proposed design. Subsequently, we used a near-field detector to obtain the electric field distribution in the *xoy* plane at *z* = 5.2 mm, including amplitude and phase information. The specific steps to follow when evaluating samples by the THz near-field scanning system can be found in Supplementary Section 6. Figure 3 depicts the simulated and measured electric field distributions of different polarization component, at the working frequency of 0.8 THz. In simulation, we chose 800 × 800 pixels as the basic profiles for constructing the electric field distribution at the focal plane, and subsequently evaluated the detection performance of the metasurface under linearly polarized incidences. The pixel coordinates occupied by the maximum value extracted from the metasurface in the *E*_*x*_-component under *x*-polarized illumination is (462, 400), and its complex amplitude can be denoted as *A*_*x*_exp(*iφ*_*x*_). Then, the complex amplitude at the same coordinates in the *E*_*y*_-component is extracted using the standard algorithm, denoted as *A*_*y*_exp(*iφ*_*y*_). As defined in the full-Stokes parameter matrix, the parameter *S*_1_ is required to distinguish between horizontal (*x*) and vertical (*y*) polarization states. To further determine the incident polarization state, the electric field distribution of the *S*_1_-component (normalized) at the focal plane is obtained visually using the calculation in Eq. (3). It can be found that the tiny spots on the focal plane with off-axis bifocal characteristics carry different polarization information. Also, the simulation field data are in good agreement with the experimental results. Subsequently, the Stokes parameter matrix of the transmitted polarization state can be rapidly reconstructed, as shown in Figure 3A. As a proof of concept, we obtained the measurements at the pixelated focal plane using the Fourier transform and extracted the corresponding complex amplitudes at the focal point by the same algorithm. It is worth mentioning that the reconstructed full-Stokes parameter matrix matches well with the simulation results, as shown in Figure 3A. In addition to the electric field intensity and section curve, here we also use the standard Poincaré sphere (PS) to intuitively show the incident polarization state. Based on the extracted measurement results, we can also plot the polarization ellipse of the transmitted polarization state for further visualization and characterization. It can be found that the reconstructed polarization trajectory is consistent with the incident polarization state. Subsequently, the electric field distributions of the *E*_*x*_- and *E*_*y*_-components at the focal plane were obtained by switching the incident mode to the *y*-polarization, as shown in Figure 3B. The acquired section curves corresponding to the electric field distribution are in agreement between the simulation and experimental results. Moreover, the distribution of the parameter *S*_1_ (normalized) gathered under *y*-polarized illumination is utilized to characterize the polarization information carried at different focal spots. It can be found that the division of the regions does not significantly affect the focusing efficiency of the metasurface [36]. The complex amplitude information at the target focal spot was extracted and its *S*-parameters were subsequently calculated using a homemade algorithm, respectively. As expected, the simulated and experimental values of parameter *S*_1_ are −1 and −0.97, respectively, while parameters *S*_2_ and *S*_3_ are both approximately equal to 0. In addition, the polarization ellipse reconstructed using the experimentally obtained amplitude and phase information completely visualizes the incident polarization mode. It is worth mentioning that we also calculated the additional parameter *DoP* (degree of polarization, defined as $DoP=\sqrt{S_{1}^{2}+S_{2}^{2}+S_{3}^{2}}/S_{0}^{2}$ ) to characterize the degree of polarization of the transmitted wave [40]. Apparently, the calculated *DoP* is 1 for arbitrary linear polarization illumination, further clarifying the reasonableness of this all-silicon polarization detector.

**Figure 4:**
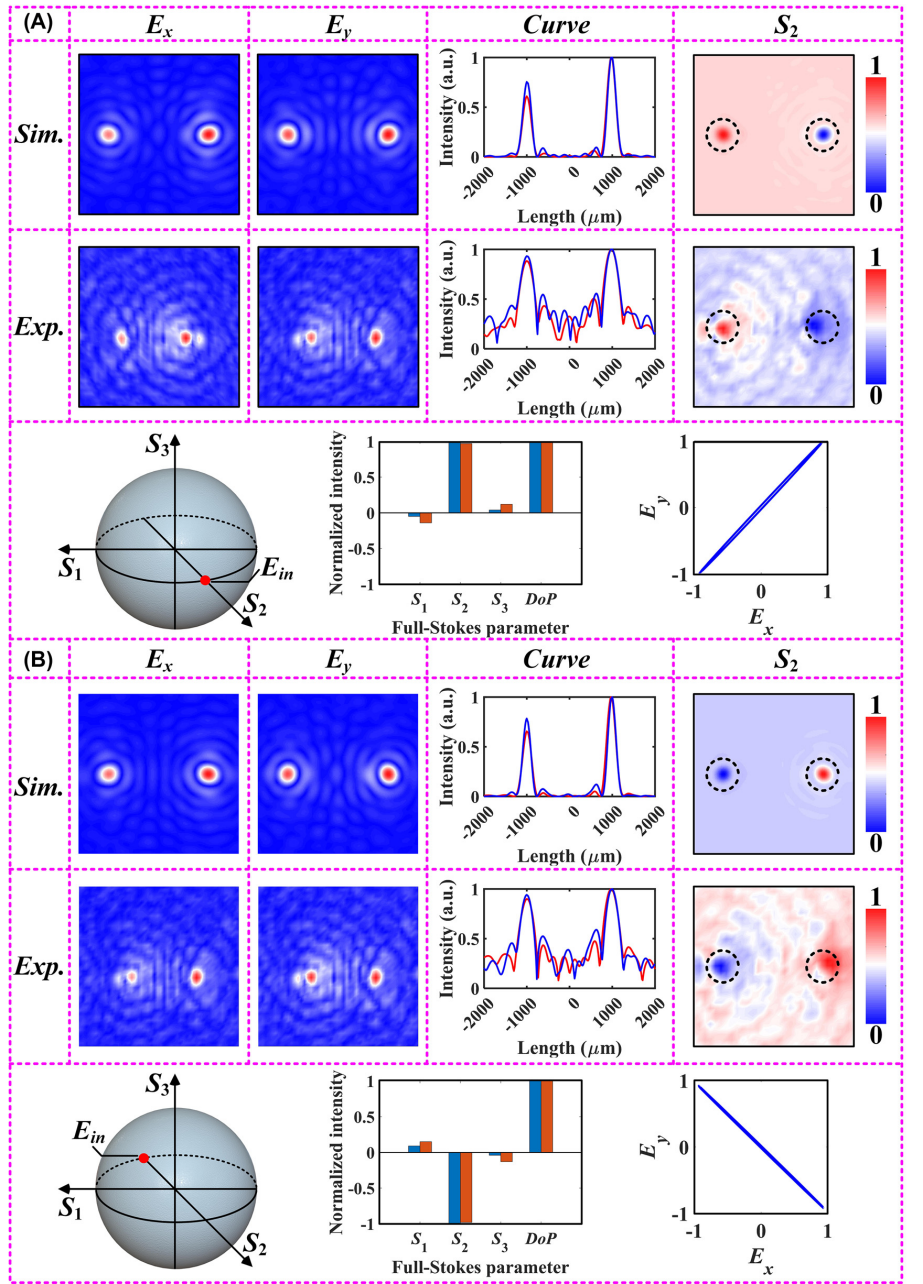
Simulated and experimental results of the fabricated samples at different polarization incidences. This includes the electric field distribution obtained from direct measurements, the normalized cross-sectional intensity profile, and the parameter *S*_2_ obtained by data processing. In addition, the incident polarization is represented using a standard PS, and the full-Stokes parameter matrix of the incident polarization and the visualized polarization ellipse are reconstructed using the complex amplitude information recorded in the focal plane. (A) Under 45°-polarized incidence, and (B) under 135°-polarized incidence.

The Jones matrix with two degrees of freedom allows for independent phase profiles to be embedded within the orthogonal polarization channel, and the transmitted polarization states must be the same as the incident ones with flipped handedness (or mutually conjugate). Therefore, when the incident polarization is switched to 45°-polarization mode, the polarization trajectory reconstructed using the extracted complex amplitude information at (462, 400) is exactly orthogonal to the incident polarization state, as mentioned in Supplementary Section 7 (see Figure S5A). In fact, due to the employment of the initial phase factor *Φ*_*ij*_, part of the incident plane wave with *x*-polarization will be artificially converted into the *E*_*y*_-component, producing a polarization conversion effect similar to the HWP. Thus, the key parameters consistent with the incident polarization state can be extracted from another coordinate (400, 337) with focusing characteristics, as shown in Figure 4A. In other words, the full-Stokes parameter matrix reconstructed at pixel point (400, 337) remains consistent with that possessed by the initially incident polarization state. The normalized intensity curves obtained from the cross-section effectively verifies the rationality of $A_{45}\exp\left(i\varphi_{45}\right)=\left(A_{x}\exp\left(i\varphi_{x}\right)+A_{y}\exp\left(i\varphi_{y}\right)\right)/\sqrt{2}$ [34, 36], i.e., with the polarization information carried by both the *E*_*x*_- and *E*_*y*_-components. In addition to the simulation and experimental results captured at the focal plane under the 45° linear polarization incidence, we also plot the profiles of the parameter *S*_2_ that determines the 45°/135° polarization state, as shown in Figure 4A. It can be clearly seen that the converging regions with off-axis distribution show different polarization distributions. Then, we evaluated the electric field distributions at the incidence of another linear polarization state that can determine the parameter *S*_2_, i.e., 135°-polarization. The utilization of a standard PS characterizes the incident polarization state, as shown in Figure 4B. The electric field distribution in the focal plane was obtained by employing a near-field probe, as shown in Figure 3D. Obviously, the experimental and simulation results obtained under 135°-polarization incidence match well. Moreover, the full-Stokes parameter matrix and the polarization trajectory reconstructed from the complex amplitudes have tolerable errors. Besides, the distribution of the normalized parameter *S*_2_ is completely opposite to the results of the metasurface under 45°-polarized illumination. Reconstruction results with the same flip characteristics can be found in Figure S5B (see Supplementary Section 7) when the incident plane waves are 135°-polarized.

**Figure 5:**
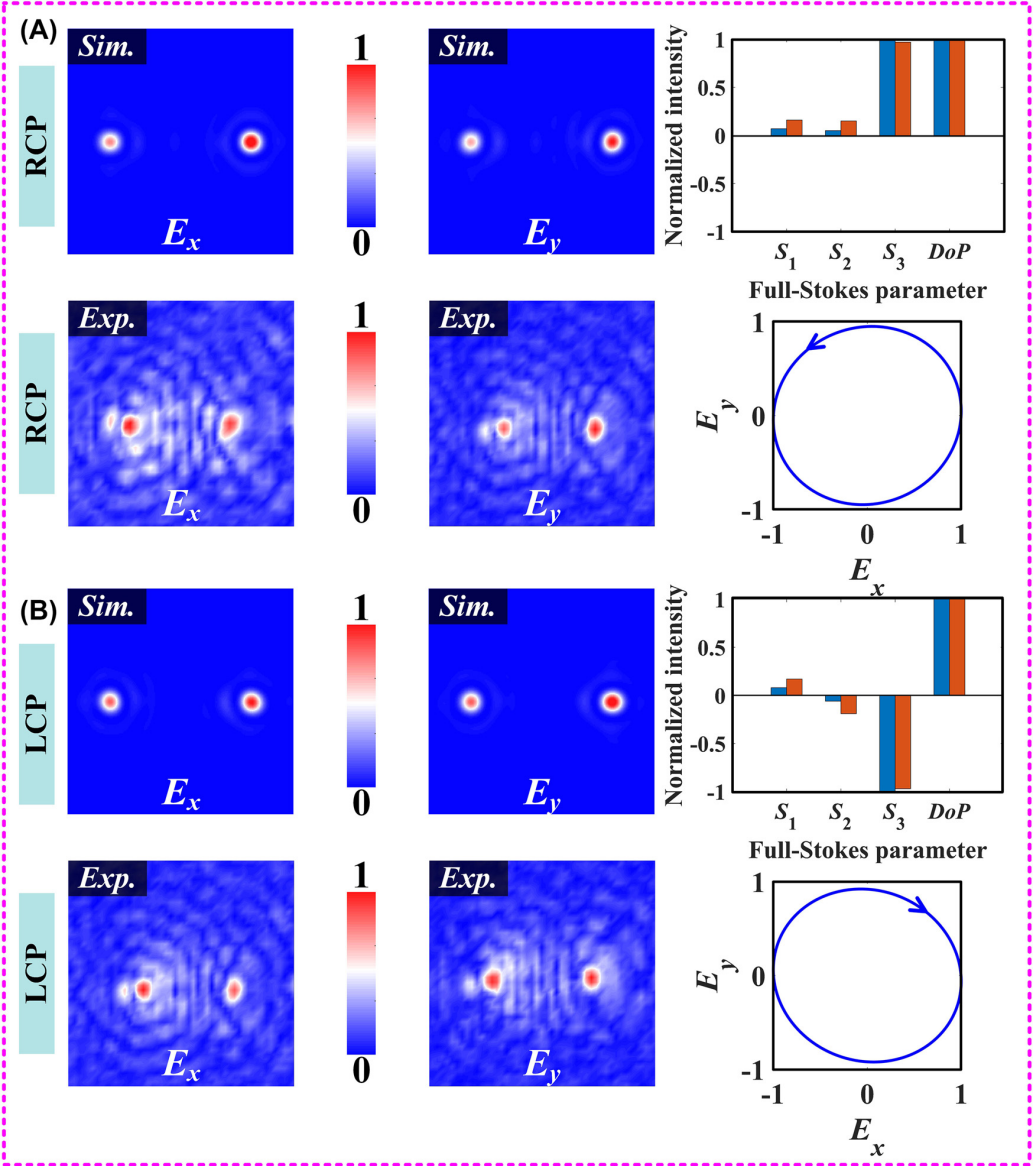
Simulated and experimental results under orthogonal CP incidence obtained using polarization conversion. The *E*_*x*_- and *E*_*y*_-components of the monitored electric field distribution are included, as well as the full-Stokes parameter matrix reconstructed from the extracted complex amplitudes on the pixelated focal plane and the polarization ellipse. (A) Under RCP incidence, and (B) under LCP incidence.

Relying on both resonant and Pancharatnam-Berry mechanisms, our meta-platform can converge THz beams with specific polarization states at different spatial coordinates and then reconstruct the full-Stokes parameters using the complex amplitude information generated by the pixelated focal plane. Unfortunately, our experimental step does not allow direct measurements of the electric field distributions of circularly polarized components. Transforming the measured data to circular-polarization electric field components $A_{\sigma }\exp\left(i\varphi_{\sigma }\right)=\left(A_{x}\exp\left(i\varphi_{x}\right)-i\sigma A_{y}\exp\left(i\varphi_{y}\right)\right)/\sqrt{2}$ (*σ* = ±1), we then observe the electric field and complex amplitudes for transmitted RCP and LCP components. Therefore, we selected orthogonal circular polarizations with opposite spins to illuminate the metasurface and monitored the *E*_*x*_- and *E*_*y*_-components at the focal plane, as shown in Figure 5. Clearly, the *E*_*x*_- and *E*_*y*_-components under RCP illumination has a bifocal characteristic with two focal spots located at pixel points (462, 400) and (337, 400), respectively. The simulation results at coordinates (462, 400) are selected as the origin of the reconstructed full-Stokes parameter matrix, as shown in Figure 5A. There is good agreement between the experimental and simulation results obtained at the same scale, and the relative error of parameter *S*_3_ is only 0.04. In addition, the plotted polarization ellipses with visualization characteristics meet the expected design requirements. In fact, the design principle relies on polarization multiplexing techniques with isolation characteristics at circularly polarized incidence. As expected, the experimental result for *S*_3_ is −1, while *S*_1_ and *S*_2_ are 0.12 and −0.14, respectively, when the proposed design is illuminated with LCP plane waves, as shown in Figure 5B. In addition, the polarization ellipse recorded according to the pixel coordinates corresponds to its reconstructed parameter matrix. Thus, the proposed all-silicon meta-platform can directly determine the polarization state carried by the incident pulsed THz wave in a single snapshot.

**Figure 6:**
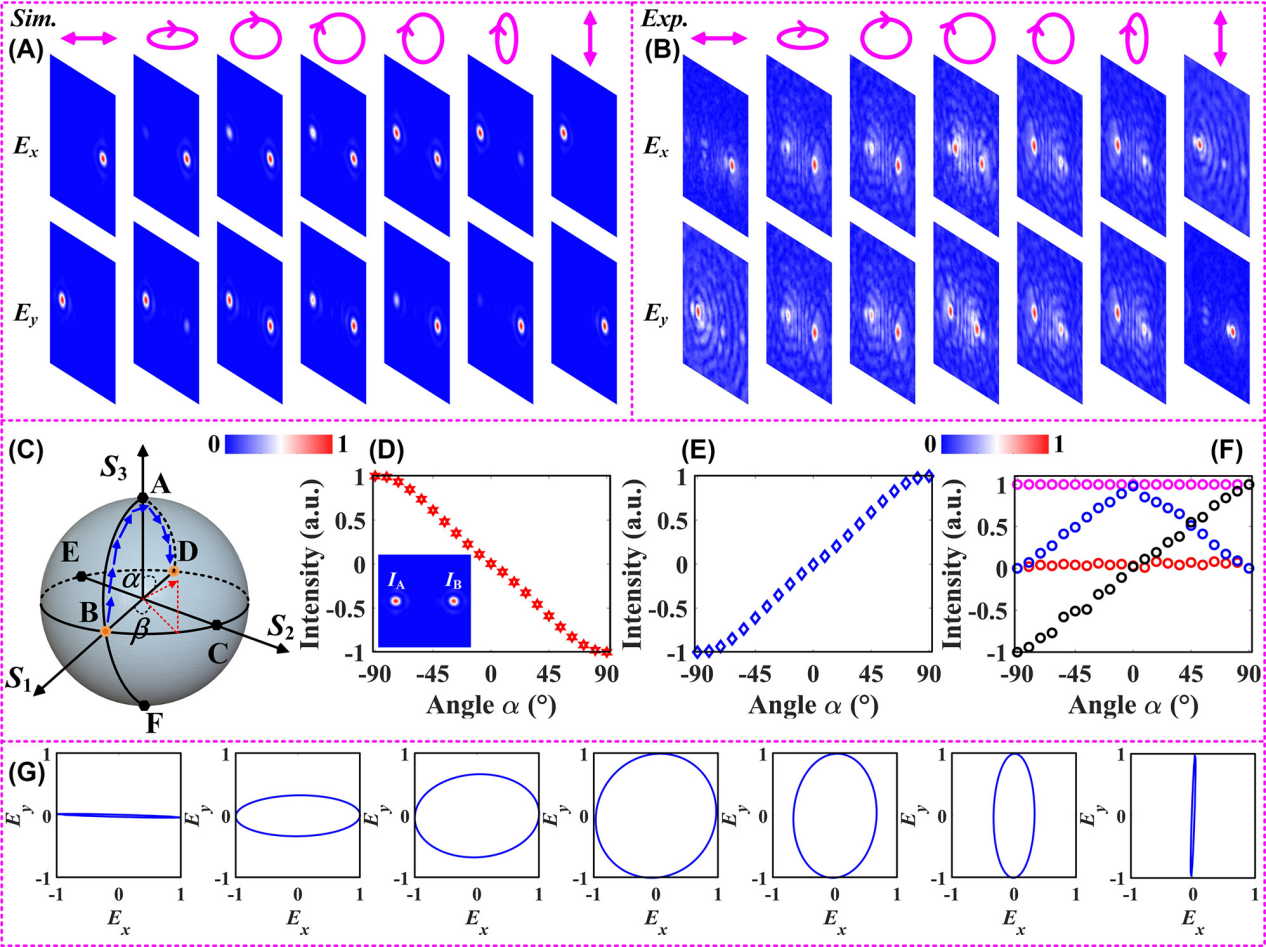
The parametric model established by using the monitored electric field distribution when the incident polarization moves along the pre-designed trajectory B → A → D. The distribution of the electric field generated in the focal plane by the incident polarization with different ellipticity can be calculated as: $\left(\cos n/m\pi \cdot \left|L\right\rangle +\sin n/m\pi \cdot \left|R\right\rangle \right)/\sqrt{2}$ , and *m*, *n* denotes an arbitrary non-zero positive integer. (A) Simulated and (B) experimental results of the electric field distributions obtained under different polarization incidence. (C) Schematic diagram of the trajectory of the incident polarization state on the surface of PS. The functions established using the intensity ratio monitored in the focal plane as the parameter *α* (D) the *E*_*x*_-component, and (E) the *E*_*y*_-component. (F) The variation curves of the *S*-parameters, and (G) polarization ellipse recorded during the evolution of the polarization state.

The above derivation procedure covers the six fundamental polarization states and profoundly demonstrates the reconstruction capability of the proposed meta-platform with off-axis bifocal characteristics for the full-Stokes parameter matrix of the incident polarization. As an example of the complex amplitude recorded on a predesigned focal plane at *x*-polarized incidence, we illustrate the broadband functional properties (≈100 GHz) of the designed metasurface in Figure S6 (Supplementary Section 8). To further evaluate the resolvability of this design mechanism for the incident polarization, we selected the random polarization located at the surface of the standard PS as the incident polarization to develop a generalized parametric theoretical model, as shown in Figure 6 [41]. The moving trajectory of the incident polarization state defined on the surface of the PS is consistent with B → A → D, i.e., the gradual conversion from *x*-polarization to *y*-polarization incidence without any change in handedness, as displayed in Figure 6C. From Figure 6A, it can be derived that the two focused spots produced in the plane of *z* = 5.2 mm are different under general polarization incidence with different ellipticity. Measurements under linearly polarized illumination were obtained using a near-field scanning system equipped with a microprobe. Subsequently, we obtained the electric field profiles under different polarization incidence using the polarization conversion step in the THz band, as shown in Figure 6B. It is worth mentioning that a good agreement is shown between simulation and experimental results. The slight difference between experiment and simulation is due to the imperfection of the sample and measurement error. As schematically shown in the inset in Figure 6D, the electric field intensities of the focal spots located in the left and right regions in the *E*_*x*_-component, defined as *I*_*A*_ and *I*_*B*_, were extracted separately. In order to evaluate more conveniently the evolutionary trend of the incident polarization state, the key parameter *η* of the incident THz beam can be calculated by the intensity ratio, that is,

(6) $$\eta =\frac{\sqrt{\left(I_{B}-I_{A}\right)/2}}{\sqrt{\left(I_{B}+I_{A}\right)/2}}$$

Figure 6D shows the experimental results obtained when the incident waves with different ellipticity are illuminated vertically on the designed metasurface. It can be found that the values of *η* are −1, 0, and 1 for the corresponding polarization states of *x*-polarization, RCP, and *y*-polarization, respectively. In other words, the employment of the parameter *η* ensures that the intensity distribution extracted in the focal plane establishes a one-to-one correspondence with the incident polarization. As the incident polarization state moves along the defined trajectory over the surface of the PS, the angle *α* gradually increases from −90° to 90° and the corresponding key parameter *η* gradually evolves from 1 to −1. As a corroboration, the off-axis bifocal characteristics exhibited in the *E*_*y*_-component are exactly opposite to those in the *E*_*x*_-component, as shown in Figure 6E. Therefore, by extracting the normalized intensity values *I*_*A*_ and *I*_*B*_ in the pixelated *E*_*y*_-component, the established functional relationship between the parameter *η* and the angle *α* exhibits an incremental evolution trend, as shown in Figure 6G. As far as we know, a completely polarization state can be described by a polarization ellipse, and the three key parameters that determine the polarization ellipse are the main axis, ellipticity, and handedness. Therefore, we can further visualize the incident polarization state by recording the complex amplitude information at the target coordinates to restore the full Stokes parameters. The schematic evolutionary behavior containing the *S*-parameters is shown in Figure 6F. The parameter *S*_1_ represented by the black circle gradually increases from 1 to −1 as the polarization state defined on the standard PS surface moves from *x*-polarization to *y*-polarization along the path B → A → D. Meanwhile, the parameter *S*_2_ remains almost constant (≈0) during the movement of the incident polarization state because the axes represented by *S*_1_ and *S*_2_ are mutually perpendicular. Moreover, since the pre-defined moving paths are concentrated in the upper part of the standard PS, the parameter *S*_3_ first experiences a gradual increase behavior from 0 to 1 and then decreases to 0. In fact, this is an evolutionary behavior in which the handedness will not be reversed. The polarization ellipse in transmission mode was reconstructed using the complex amplitude information carried by the pixelated focal, as shown in Figure 6G. Benefiting from the appropriate step size of the microprobe when moving, the plotted polarization trajectories match well with the theoretical distribution (Figure 6).

## 4 Conclusions

In summary, a facile all-silicon metasurface mechanism operating in the THz band is demonstrated that allows direct polarization detection for the incident plane waves. The focal spots on the pre-designed focal plane for characterizing the polarization state has off-axis bifocal characteristics based on the polarization multiplexing phase encoding technique. Recording the electric field distribution in the focal plane one by one by employing microprobes allows us to reconstruct the full Stokes parameter matrix of the incident polarization state using standard algorithms. The simulation results for the typical six polarization incidence modes obtained by the time-domain finite integration method show good agreement with the experimental results. Subsequently, the evolutionary trend of the spot with off-axis bifocal properties on the focal plane was evaluated by defining a random incident polarization state on the surface of a standard PS. Moreover, the trajectory of the incident polarization under a predetermined path is visualized using polarization ellipses to further confirm the polarization detection capability of this meta-platform. We expect the proposed mechanism to be of considerable interest for applications in polarimetric THz imaging, communication, and remote sensing.

## Supplementary Material

Supplementary Material Details
