# Supplementary material for: Terahertz all-silicon metasurfaces with off-axis bifocal characteristics for polarization detection
Source: Nanophotonics. 2023 Jun 22;12(16):3359–71. doi: 10.1515/nanoph-2023-0277 (PMC11501994; doi:10.1515/nanoph-2023-0277)
Supplement: Supplementary file 1 — Supplementary Material Details [file j_nanoph-2023-0277_suppl_001.docx]

**Supplementary Information**

Terahertz all-silicon metasurfaces with off-axis bifocal characteristics for polarization detection

Hui Li,^a^ Shouxin Duan,^b^ Chenglong Zheng,^a^ Hang Xu,^a^ Jie Li,^a,c^ Chunyu Song,^a^ Fan Yang,^a^ Wei Shi,^a,*^ Yating Zhang,^a,*^ Yun Shen,^b,*^ Jianquan Yao,^a,*^

a Key Laboratory of Opto-Electronics Information Technology (Tianjin University), Ministry of Education, School of Precision Instruments and Opto-Electronics Engineering, Tianjin University, No. 92 WeiJin Road, Tianjin 300072, China

b Department of Physics, School of Physics and Materials Science, Nanchang University, Nanchang 330031, China

c Information Materials and Device Applications Key Laboratory of Sichuan Province, Chengdu University of Information Technology, Chengdu, China, 610225

*Address all correspondence to Wei Shi: [shiwei@tju.edu.cn](mailto:shiwei@tju.edu.cn); Yating Zhang: yating@tju.edu.cn; Yun Shen: shenyun@ncu.edu.cn; Jianquan Yao: jqyao@tju.edu.cn

1. **The generalized design solution is demonstrated theoretically at 10.6 µm**

To the best of our knowledge, the spin-polarization multiplexing coding technique is universal and can be applied to metasurface designs operating at arbitrary wavelengths [Ref. 1]. To further evaluate the superiority of the proposed design principle, we plot in Figure R3 the electric field distribution at the focal plane through the same encoding approach for a metasurface working at 10.6 μm. The selected meta-atoms are still in all-silicon configuration. And, the height of the rectangular column with anisotropy is H = 6 μm [Ref. 2]. Moreover, meta-atoms that meet the constraints of Equation 1 were selected with a phase interval of 45°, and their electric field distribution at six different polarization incidence is shown in Figure S1. Thus, with the original electric field data at different polarization incidence, we can then further analyze its *S*-parameters. In other words, the full-Stokes parameter matrix can be reconstructed by monitoring the complex amplitudes carrying polarization information at the focal plane. This also reflects the general applicability of the proposed design principles.


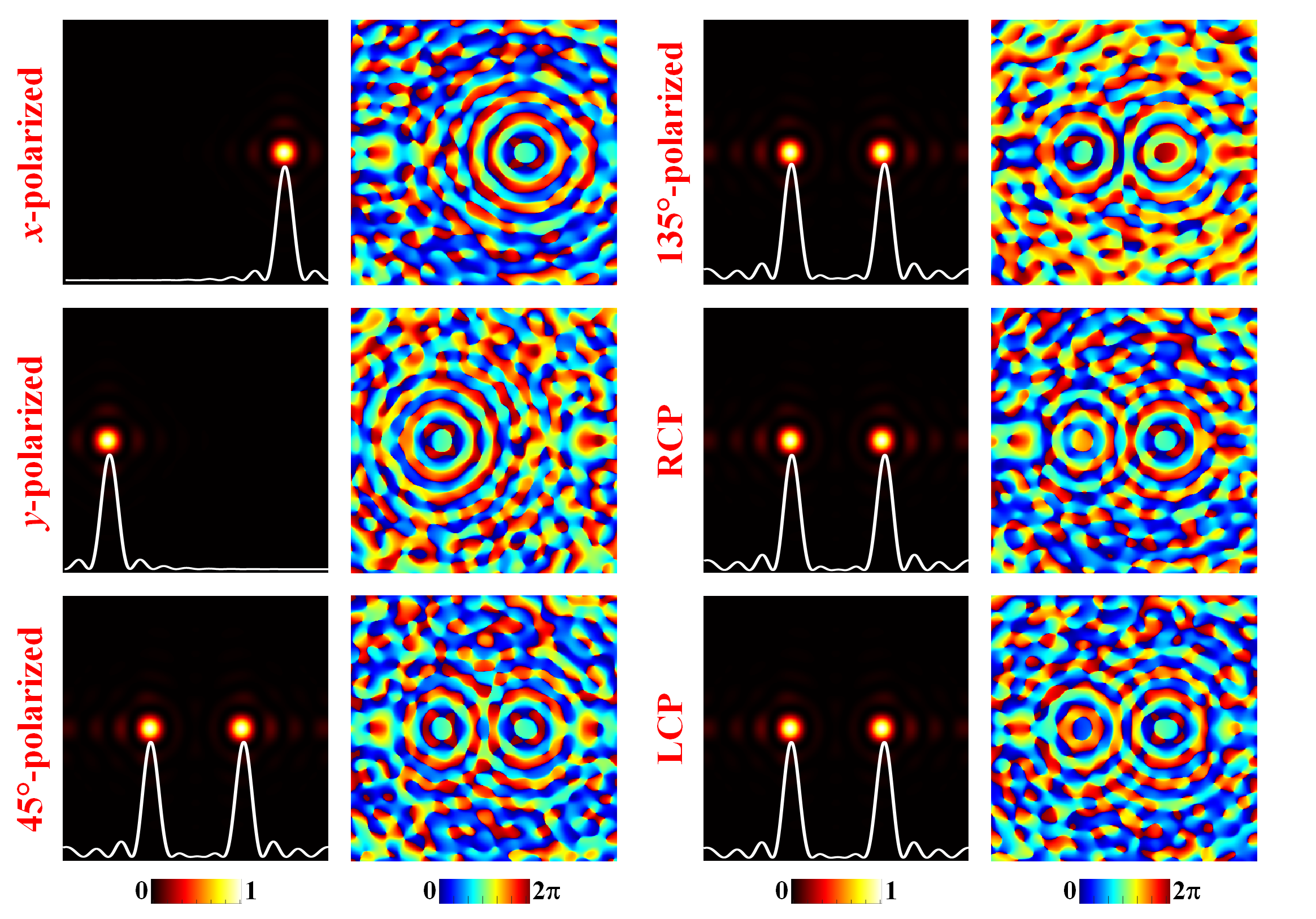


**Figure S1.** The electric field distribution generated by all-dielectric metasurfaces operating at 10.6 µm in a pre-designed focal plane.

1. **Numerical Simulations**

The polarization behavior of the all-dielectric basic building blocks that comprise the decoupled metasurface is performed by using the time-domain finite integration solver of the CST Microwave Studio software. The basic computational model of the time-domain finite integration algorithm in CST is mainly based on the spatial discretization method, the corresponding Maxwell’s lattice equations generated can be expressed as,

(A1)

After these steps, the integral equation can be converted to a linear equation for solution and the electromagnetic parameters of the target structure can be obtained directly in the calculation. The amplitude and phase response of meta-atoms under orthogonal linear polarization incidence can be obtained directly using the post-processing template of the CST software. By scanning the polarization information of the building blocks over a given range of parameters, the fundamental database can be established for the selection of meta-atoms that satisfy the constraints of Equation (3). To obtain the far-field distribution as shown in Figure 1, we still perform the calculation by employing the CST software. The boundary conditions in the *x*, *y* and *z* directions are set to OPEN, and the added field monitor is placed at a distance greater than the focal distance from the metasurface to evaluate the electric field distribution at the focal plane in detail.

1. **Response of meta-atoms similar to a HWP supported by FP-resonance**


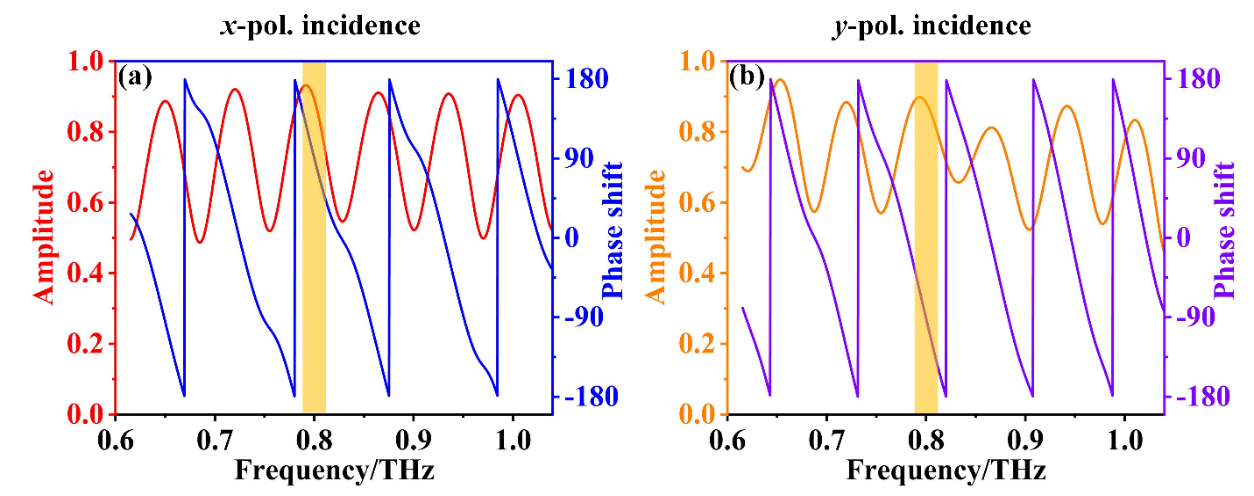


Figure S2. Stimulated spectra of transmission amplitude and phase of the designed metasurface under (a) *x*-polarized and (b) *y*-polarized incidence, respectively.

As an example, the amplitude and phase response at 0.8 THz of the basic building block with structural parameters *L*_1_=23 and *L*_2_=114 are shown in Figure S2. With the illumination of THz plane waves with *x*(*y*) polarization, the amplitude of the co-polarized component at 0.8 THz is nearly 90%. In addition, the selected meta-atoms have a phase difference of π at orthogonal linearly polarized incidence (*p_xx_*=90.52, *p_yy_*=-91.15). The oscillations shown in the transmission spectrum can be qualitatively interpreted as FP-resonances.

1. **The structural parameters of the meta-atoms selected as candidates**

**Table 1:** The structural parameters of the selected meta-atoms.

| No. | 1 | 2 | 3 | 4 | 5 | 6 | 7 | 8 | 9 | 10 | 11 | 12 | 13 | 14 | 15 |
| --- | --- | --- | --- | --- | --- | --- | --- | --- | --- | --- | --- | --- | --- | --- | --- |
| *L*_1_ | 84 | 85 | 88 | 93 | 114 | 57 | 55 | 52 | 49 | 45 | 40 | 34 | 23 | 87 | 85 |
| *L*_2_ | 49 | 45 | 40 | 34 | 23 | 87 | 85 | 85 | 84 | 85 | 88 | 93 | 114 | 57 | 55 |
| *t_xx_* | 0.787 | 0.825 | 0.856 | 0.883 | 0.883 | 0.839 | 0.816 | 0.815 | 0.838 | 0.871 | 0.888 | 0.893 | 0.893 | 0.781 | 0.752 |
| *t_yy_* | 0.838 | 0.871 | 0.888 | 0.893 | 0.893 | 0.781 | 0.752 | 0.754 | 0.787 | 0.823 | 0.856 | 0.883 | 0.883 | 0.839 | 0.816 |
| *p_xx_* | -178.2 | -156.9 | -137.6 | -114.3 | -91.2 | -69.1 | -46.4 | -22.5 | -0.6 | 22.5 | 45.1 | 65.9 | 90.5 | 110.8 | 138.1 |
| *p_yy_* | -0.6 | 22.5 | 45.1 | 65.9 | 90.5 | 110.8 | 138.1 | 155.8 | -178.2 | -156.9 | -137.6 | -114.3 | -91.2 | -69.1 | -46.4 |

The structural parameters of the basic building blocks that conform to the principle of phase decoupling searched using the homemade algorithm are shown in Table 1. It can be found that the maximum aspect ratio (AR) is about 17:1, which is in accordance with the processing accuracy of the system.

1. **Sample Fabrication**

The proposed all-silicon metasurface is fabricated by standard photolithography and deep reactive ion etching technology. Highly resistive silicon wafers with a refractive index of 3.45 having a high transmittance efficiency (≈0.7) were selected as candidates for fabrication of the desired samples. The fabrication step can be found in Figure S3. In the first step, a chromium layer with a thickness of ≈10 nm is sputtered on a double-sided polished silicon wafer with a diameter of 4 inches and a thickness of 1 mm. In the second step, an approximately 10-μm-thick layer of AZ2070 photoresist is spin-coated on the target hybrid silicon wafer. Fabrication errors on the order of micrometers led us to decide to use a custom mask. Subsequently, the photoresist was precisely conformed and developed by conventional UV lithography. The chromium layer not protected by the photoresist then needs to be removed by acid etching. Subsequently, the desired sample was etched by employing (ICP) etching technique (STS MULTIPLEX ASE-HRM ICP ETCHER, UK) with an etching depth of 400 μm. Finally, the desired all-silicon metasurface was obtained by cleaning the photoresist and chromium layer remaining on the sample surface using acetone and acid, respectively.


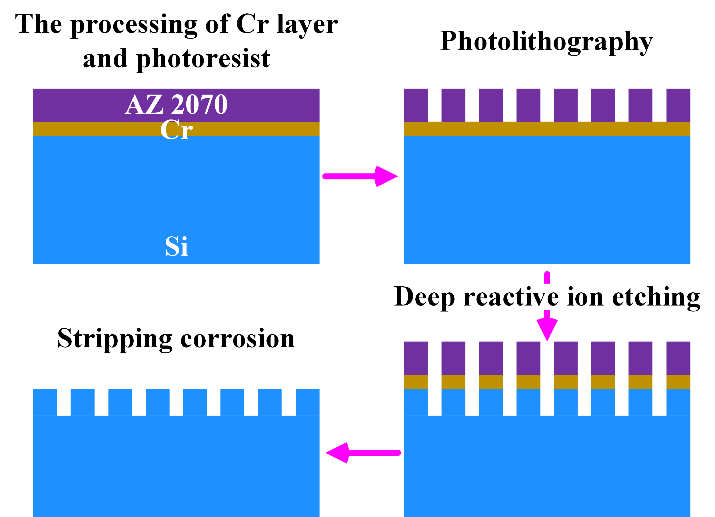


Figure S3. The flow diagram of sample fabrication.

1. **Experimental Characterization**

The electric field distributions of the fabricated all-silicon sample were measured by employing a THz near-field detection system (TeraCube Scientific M2), and the probe was placed at a distance of about 5 mm above the sample, as shown in Figure S4. The femtosecond laser source in the constituent near-field scanning system is 780 nm with a pulse width of 100 fs and a repetition frequency of 80 MHz. The microprobe scans the focal plane of the sample in steps of 60 μm and records the complex amplitude information on the focal plane pixel by pixel. The initial electric field distributions of the *Ex*- and *Ey*-components were obtained by replacing the probe and rotating the sample, respectively. Subsequently, the information on the complex amplitudes under different polarization illumination was obtained using the conversion relation between each polarization state. It should be mentioned that both the simulation and the experimental results are processed using a homemade algorithm.


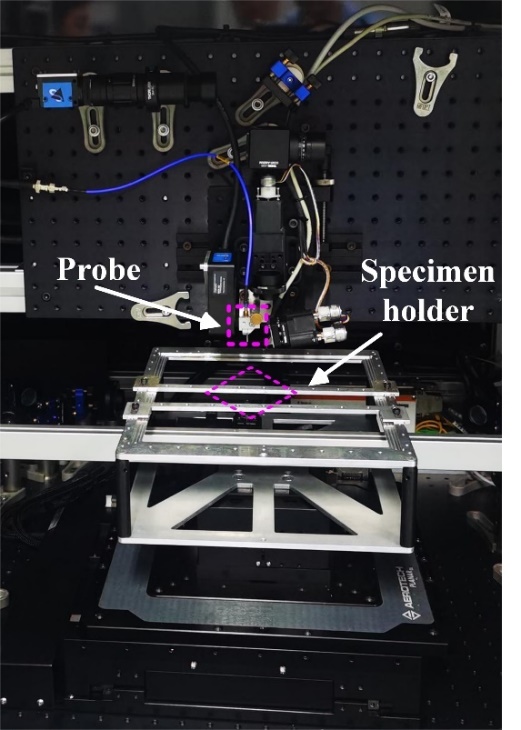


Figure S4. Schematic of the THz near-field scanning time-domain spectroscopy system, near-field probe with ≥ 60 μm resolution.

1. **Polarization ellipse reconstructed at coordinates (462, 400) under 45°/135° polarization illumination**


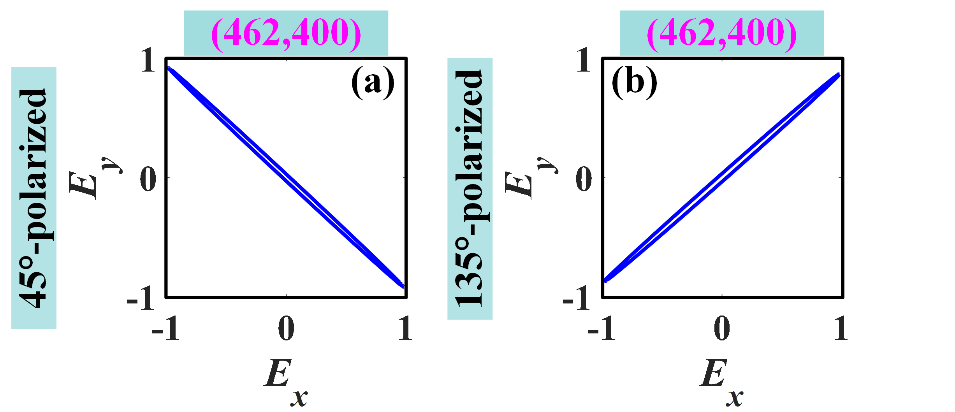


Figure S5. The reconstructed polarization ellipse images at pixel coordinates of (462, 400) under (a) 45°-polarization and (b) 135°-polarization illumination, respectively.

Obviously, the direction of polarization characterized by the polarization ellipse reconstructed at coordinates (462, 400) under 45°/135° polarization illumination is perpendicular to the incident polarization.

1. **Broadband performance of the designed metasurface under *x*-polarized illumination**


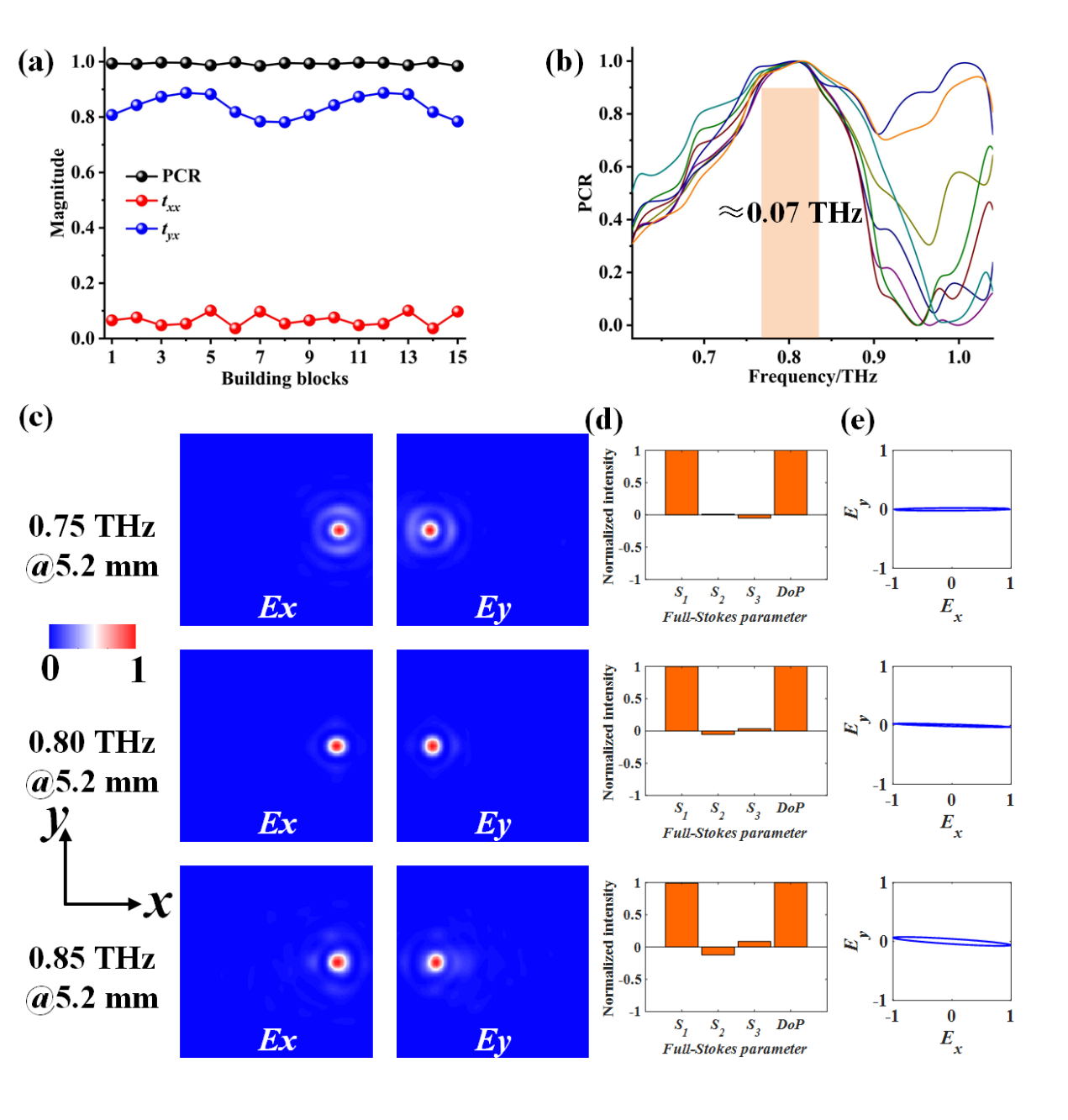


Figure S6. (a) Transmission amplitude and PCR of the selected basic building blocks at 0.8 THz. (b) PCR spectra of the 15 basic building blocks in the broadband range. The (c) electric field distribution, (d) full Stokes parameters and (e) polarization ellipse monitored at 0.75 THz, 0.8 THz, and 0.85 THz, respectively, using the simulation software when the focal length is fixed at *z* = 5.2 mm.

Due to the limitations of the spin-polarization multiplexing coding technique, the behavior of the selected basic building blocks strictly follows that of the perfect half-wave plate. Therefore, we first evaluated the polarization conversion ratio (PCR) of 15 meta-atoms in the broadband range. As can be seen in Figures S6a and S6b, the meta-atom with an all-silicon configuration has good polarization conversion performance at 0.8 THz. In addition, an operating bandwidth of about 0.07 THz (PCR≥90%) exists for the basic building block. As shown in Figures S6d-e, the focal length of the metasurface operating at 0.8 THz was fixed at 5.2 mm, and we reconstructed the full-Stokes parameters of the incident polarization state as well as the polarization ellipse at 0.75 THz, 0.80 THz, and 0.85 THz, respectively. Obviously, the obtained results can effectively prove that the incident polarization state is *x*-polarized. In other words, the proposed metasurface based on all-silicon configuration has a comparable operating bandwidth.

***References***

[Ref. 1] Phys. Rev. Lett. 118, 113901 (2017).

[Ref. 2] Adv. Funct. Mater. 27, 1704295 (2017).
